# Domain-specific contexts promote model-based decision making for basketball players

**DOI:** 10.1038/s41598-026-54649-z

**Published:** 2026-06-18

**Authors:** Liu Yang, Chenglin Zhou, Biao Sang

**Affiliations:** 1 School of Psychology, Shanghai University of Sport, No.399, Changhai Road, Yangpu District, Shanghai, China; 2 Lab for Educational Big Data and Policymaking, Shanghai Academy of Educational Sciences, Shanghai, China

**Keywords:** Reinforcement learning, Expertise, Two-stage task, Model-based, Model-free, Psychology, Human behaviour

## Abstract

Decision-making is a critical component of basketball performance. Within the framework of Reinforcement Learning (RL), understanding the environment and task structure can facilitate model-based (MB) behavior. This study tested whether domain-specific contexts promote MB decision strategies by using the two-stage task in a 2 (Group: basketball players vs. novices) × 2 (Condition: abstract symbols vs. basketball tactical diagrams) mixed design. Thirty-five national basketball players and twenty-seven novices completed both conditions. A hierarchical one-trial-back logistic regression estimated intercept, reward, transition, and reward × transition coefficients; two-factor repeated-measures ANOVAs assessed coefficients, reaction times, and subjective evaluations. Compared to novices, basketball players exhibited a more MB decision-making strategy across all condition, accompanied by higher subjective understanding. Novices exhibited higher reward coefficients indicated a more model-free (MF) tendency. For basketball players, MB strategies were associated with longer RTs, yet longer RTs in novices did not necessarily indicate MB strategies use. These findings show that expertise facilitates the integration of outcome feedback with the task’s structure, thereby leading to MB decision-making. Incorporating sport-specific displays into training may enhance decision-making performance without relying on explicit structural instruction.

## Introduction

Decision-making is a critical component of basketball performance, and the quality of decisions in clutch moments often dictates the outcome of the game. Human decision-making behaviors are commonly categorized into goal-directed (model-based, MB) and habitual (model-free, MF) processes in the reinforcement learning (RL) framework^1,2^. Decision-making model refers to a computational framework grounded in RL to represent the underlying cognitive mechanisms of decision behavior. The MF strategy leads to repeats past actions that frequently resulted in rewards, whereas the MB strategy relies on a mental model of the environment and psychological representation of the task to plan and maximize rewarding outcomes^3,4^. As researchers continue to delve deeper into the field of RL decision-making behavior, findings reveal that humans use not only MF or MB strategies in a binary manner, but also hybrid and other models^5^.

The expertise (i.e., the domain-specific perceptual-cognitive advantage developed through prolonged and systematic basketball practice) enhances athletes’ ability to make rapid, accurate, and strategic decisions^6,7^. Particularly in team sports such as basketball, expert decision-making involves integrating perceptual, cognitive, and motor processes dynamically, enabling adaptive responses to evolving competitive contexts^8–12^. In the ecological dynamics approach, emphasis is placed on the role of expertise in attuning athletes to affordances and on the dynamic interactions between the athlete and the environment to guide action^13,14^. For instance, the outcome of actions influences subsequent planning; basketball players are more likely to attempt another three-point shot if their last attempt was successful^15^. These findings align with the RL perspective, which emphasizes that the basketball players adjust decisions based on prior outcomes and expertise. Although given the complexity of identifying the influences of expertise on the continuous decision-making process in basketball, the two-stage task emerges as a critical tool. It stands as the sole optimal paradigm capable of dissociating between MF and MB^16^.

The critical feature of the two-stage task is that it requires participants to go through two sequential stages in each trial. In the task, participants make a binary choice at the first stage of each trial and then transitioned probabilistically to different second-stage states^16,17^. In the second stage, depending on the other binary choice, they may (or may not) receive a reward, the reward probabilities of these second-stage options change throughout the task. One-trial-back logistic regression is needed to adopted because it consistently provides the best fit among commonly used models in the two-stage task^17^. The MF strategy focuses on repeating previously rewarded actions regardless of transition, whereas the MB strategy involves a more comprehensive consideration of the task, leading to a larger reward × transition interaction effect^5,18–21^.

Transforming the abstract instructions into a narrative involving a magic carpet or spaceship story led healthy adult humans to exhibit behavior more characteristic of MB decision-making, as the storytelling-based instructions enhanced their understanding and reduced effort with the two-stage task by leveraging the natural human affinity for stories to facilitate deeper cognitive processing and strategic thinking^17^. This concept mirrors the phenomenon whereby experts achieve greater success in domain-specific contexts. Because they possess a better understanding of these areas and engage in more in-depth planning for the future, employing strategies more characteristic of MB decision-making^22,23^. Thus, the two-stage task with different conditions (abstract condition vs. basketball condition) may provide a foundational understanding of why strategies can shift from MF to MB, which is relevant to understanding more complex decision-making environments and the role of expertise.

In summary, previous research has found that expertise enhances decision-making performance in specific domains, but it has not thoroughly uncovered the underlying mechanisms and reasons^24,25^. The ongoing development of the RL framework in the field of general psychology provides strong theoretical and methodological support for this phenomenon. This study seeks to bridge this gap by examining how skilled basketball players’ decision-making strategies vary between conditions that involve basketball-specific and abstract materials. Therefore, the present study aimed to use the two-stage task to determine whether experts in domain-specific contexts employed a more MB decision-making strategy. Furthermore, we will also investigate the reaction time and subjective evaluations (i.e., understanding, effort, complexity) of participants during task execution to better explain the reasons behind behavioral changes^17^. Based on previous findings^13,17^, we hypothesized that: (1) Basketball players would exhibit a more MB strategy (i.e., larger reward × transition interaction effect) in basketball-specific contexts compared to abstract contexts. (2) Because basketball tactical diagrams contain a greater amount of information, participants would to exhibit slower reaction times compared to abstract contexts. (3a) Basketball players would report better understanding of the task in basketball-specific contexts than in abstract contexts. (3b) Basketball players would put less effort in basketball-specific contexts than in abstract contexts. (3c) Basketball players would not perceive the task as less complex in basketball-specific contexts than in abstract contexts.

## Materials and methods

### Ethic statement

The studies involving humans were approved by the Sports Science Experiment Ethics Committee of Shanghai University of Sport (No. 102772024RT074). The studies were conducted in accordance with the local legislation and institutional requirements. All participants provided informed consent before participation.

### Participants

The sample size was determined based on the recommendations of Brysbaert (2019)^26^ and estimated using G*Power 3.1^27^. A repeated measures ANOVA was selected, with the parameters set as effect size *f* = 0.5, *α err prob* = 0.05, *power (1-β err prob)* = 0.95. The calculation results indicated that a total of 54 participants would be required.

Thirty-seven national second-level basketball players participated in this experiment. Two participants were excluded from the analysis: one due to a complete lack of understanding of the task, and one due to an excessively long average response time (5 s). A total of 35 basketball players were included in the final analysis (28 males, age = 20.57 ± 1.48 years, Mean ± SD). They have been playing basketball for 9.14 ± 1.85 years (Mean ± SD) and train for 10.91 ± 4.10 h per week (Mean ± SD). The control group consisted of 27 participants (10 males), with a mean age of 22.90 ± 3.56 years. None of them had any prior experience in playing or watching basketball. All the participants had normal or corrected-to-normal visual acuity.

### Experimental design

The experiment utilized a 2 (Group: basketball players vs. novices) × 2 (Condition: abstract symbols vs. basketball tactical diagrams) mixed-design. Group was the between-subjects factor and Condition was the within-subjects factor. The dependent variables included three categories: (1) Reaction times at Stage 1 and Stage 2; (2) Estimated coefficients from the one-trial-back logistic regression analysis, including the intercept, reward, transition, and reward × transition interaction; (3) Subjective evaluations (understanding, effort, and perceived complexity) collected after completing each block.

### Two-stage task

**Fig. 1:**
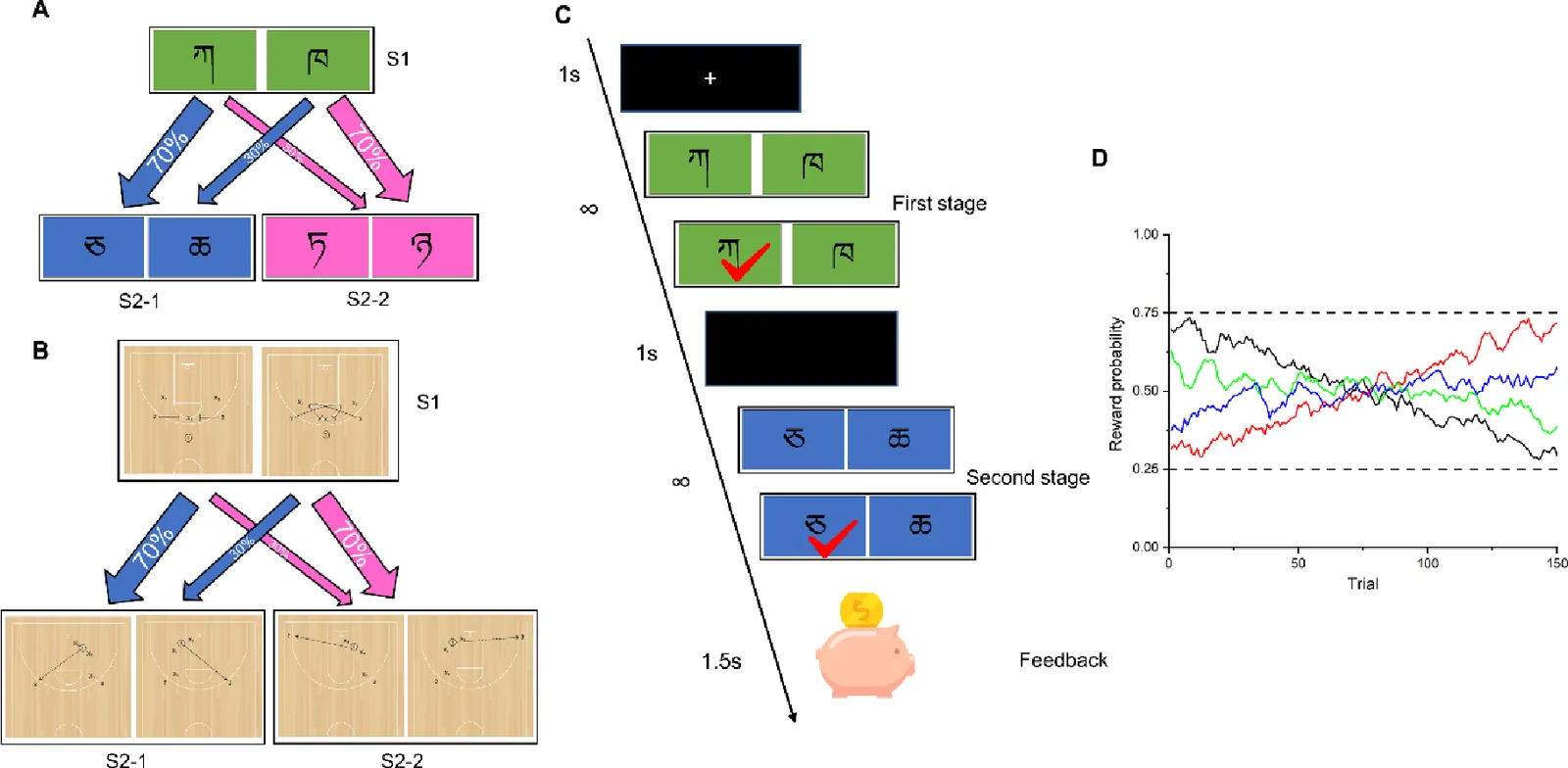
Structure and timeline of two-stage tasks. (**A**) The original two-stage task^16^ under abstract condition. In the S1, participants choose one of two green symbols. Depending on which symbol they choose, they transition to the pink state or the blue state. In the S2, participants choose one of two pink or blue symbols. (**B**) The two-stage task adapted for the basketball condition, substituting original abstract symbols with well-designed basketball tactical diagrams. (**C**) The timeline of the two-stage tasks in the present study used the same settings for both the abstract and basketball conditions. (**D**) The reward probabilities of the four S2 options change between 0.25 and 0.75 during the task. S1, stage one (i.e., the first stage); S2, stage two (i.e., the second stage); S2-1, the first options set in stage two; S2-2, the second options set in stage two.

The present task adhered to the framework of the original two-stage task (Fig. 1C) and was developed using MATLAB R2021b, incorporating Psychtoolbox extensions (version 3.0.19). At the beginning of each trial, a fixation cross was displayed for one second. Following the disappearance of the fixation cross, participants were presented with two options for Stage 1 (S1), from which they were required to make their initial decisions. After a one-second black screen, the participants proceeded to Stage 2 (S2). The stimuli presented in S2 were determined based on the decision made in S1. For instance, if participants selected option 1, there was a 70% probability of advancing to S2-1 (a common transition) and a 30% probability of advancing to S2-2 (a rare transition). In S2, participants were required to make another decision and receive feedback (either a reward or no reward). The average probability of receiving a reward for each option was 50%, with a trend of linear variation within the range of 25–75% (Fig. 1D). To avoid the effect of stimulus-response learning, we set the response option positions at random^1,18^. The task comprised two blocks: an abstract symbols block (Fig. 1A) and a basketball tactical diagram block (Fig. 1B), with each block containing 150 trials. All the program files are publicly available at https://osf.io/apkmz/?view_only=268f215e593b4f6ca11c3f04facc862f.

#### Basketball tactical diagrams and assessment

Designed in collaboration with two basketball experts, a total of six 3V3 basketball tactical diagrams serve as stimulus materials. The two diagrams in S1 represent the initiation phase of two tactics, and the two diagrams in S2-1 and S2-2 represent the completion phase of the tactic. Additionally, in the basketball scenario, each option at S1 can potentially lead to any of the four states at S2. Furthermore, the two options in both S2-1 and S2-2 were mirrored, reflecting symmetrical tactical alternatives (Fig. 1B).

To minimize decision-making bias due to the tactical significance of the diagrams as much as possible, we invited 23 basketball players to evaluate these three pairs of tactical diagrams using a 5-point Likert scale (1 = completely inconsistent, 3 = neutral, 5 = completely consistent). The scale comprises three items, each associated with a pair of tactical basketball diagrams. Evaluators were asked, ‘If the abilities of Players 2 and 3 are completely equal, do the two diagrams represent consistent tactical significance?’ In the two-stage task, the decision in S2 directly impacts rewards, and taking into account the framework of the Spaceship or Magic Carpet versions of the two-stage task^5^, we aim for our basketball tactical diagrams in S1 to be perceived as neutral (equal to 3), and those in S2-1 and S2-2 to be viewed as consistent (rated higher than 3).

### Procedure

After signing the informed consent form, the condition of the initial block for each participant was determined by using a random number generator. Subsequently, participants read the experimental instructions and completed a practice session of 15 trials before conducting the formal experiment. The experimental instructions are as follow: “Welcome to this experiment. In the first stage, you will need to make a decision between two options: press the left arrow key (←) to select the option on the left and the right arrow key (→) to select the option on the right. Each option will lead to a specific second stage with a 70% probability or to another second stage with a 30% probability. In the second stage, a decision is made between the two options: press the left arrow key (←) to select the option on the left and the right arrow key (→) to select the option on the right. This decision may or may not result in reward. Furthermore, the probability of these rewards changes over time, and you will need to continuously explore and learn to find the best decisions. Your goal is to obtain as many rewards as possible. Once you understand the intent of the experiment, press the space key to start the practice.” The participants began the formal experiment after the practice session. At the end of each block, participants were asked the following questions: (1) To what extent do you understand the task? (0 represents no understanding at all, 4 represents complete understanding); (2) How much effort did you put into completing this task? (0 = no effort, 4 = maximum effort); and (3) How complex do you find the task? (0 = very simple; 4 = very complex).

### Data analysis

Considering the potential influence of time pressure on decision-making behaviors, the present study did not impose time limits on decisions made during S1 and S2^28,29^. To ensure the analysis of typical decision-making times and to mitigate the effects of unusually quick or delayed reactions, trials with a reaction time z-score at S1 or S2 above or below 2 were excluded from the data analysis.

#### One-trial-back logistic regression analysis

We utilized Python (version 3.6.6) and the CmdStanPy package (version 1.1.0) to execute a hierarchical logistic regression model, with parameters estimated using Bayesian computational methods^17^. The predicted variable was *p*_*stay*_, representing the stay probability, and the predictors included *x*_*r*_, indicating receipt of a reward in the previous trial (+ 1 if rewarded, − 1 otherwise); *x*_*t*_, which indicated whether the transition in the previous trial was common or rare (+ 1 if it was common, − 1 if it was rare), and the interaction of the two. Thus, for each participant under both conditions, an intercept *β*_*0*_ and three coefficients were determined, as shown in the following equation:

1 $$\:{p}_{\mathrm{s}\mathrm{t}\mathrm{a}\mathrm{y}}=\frac{1}{1+\mathrm{e}\mathrm{x}\mathrm{p}[-({\beta\:}_{0}+{\beta\:}_{r}{x}_{r}+{\beta\:}_{t}{x}_{t}+{\beta\:}_{r\times\:t}{x}_{r}{x}_{t}\left)\right]}$$

For each trial pair, the variable *y* was set to 1 if the participant selected the same S1 action in the subsequent trial as in the previous trial (a “stay” choice), and set to 0 if the participant chose a different S1 action (a “switch” choice). The distribution of *y* is Bernoulli (*p*_*stay*_). Further details on the Bayesian model parameters and settings can be found in Feher da Silva et al.^17^. The model was estimated using four Markov chains, each with 32,000 iterations, including 16,000 warm-up samples, resulting in a total of 64,000 post-warm-up samples. All parameters exhibited R̂ (R-hat) values approximately equal to 1.0, indicating satisfactory convergence of the chains.

#### One sample t-test and repeated measures ANOVA

R (version 4.4.0) was used to perform all statistical analyses. One-sample t-test was conducted on the assessment of basketball tactical diagrams. For all other dependent variables, a two-factor repeated measures ANOVA was conducted, with Group (basketball players vs. novices) as the between-subject variable, and Condition (abstract symbols vs. basketball tactical diagrams) as the within-subject variables. The effect sizes were measured using generalized eta squared, which represents the proportion of the total variance accounted for by the effect.

## Results

### Basketball tactical diagrams assessment

The one-sample t-test indicated that evaluators perceived the diagrams in S1 was equal to 3 (*t* = 1.29, *df* = 22, *p* = 0.211, 95% CI [2.74, 4.14]). Conversely, for diagrams in S2-1 (*t* = 9.71, *df* = 22, *p* < 0.001, 95% CI [4.16, 4.79]) and S2-2 (*t* = 15.46, *df* = 22, *p* < 0.001, 95% CI [4.39, 4.82]), there was extremely strong evidence suggesting that evaluators considered them consistent in terms of tactical significance (higher than 3). These results align with our expectations for basketball tactical diagrams.

### Coefficients of one-trial-back logistic regression model

**Fig. 2:**
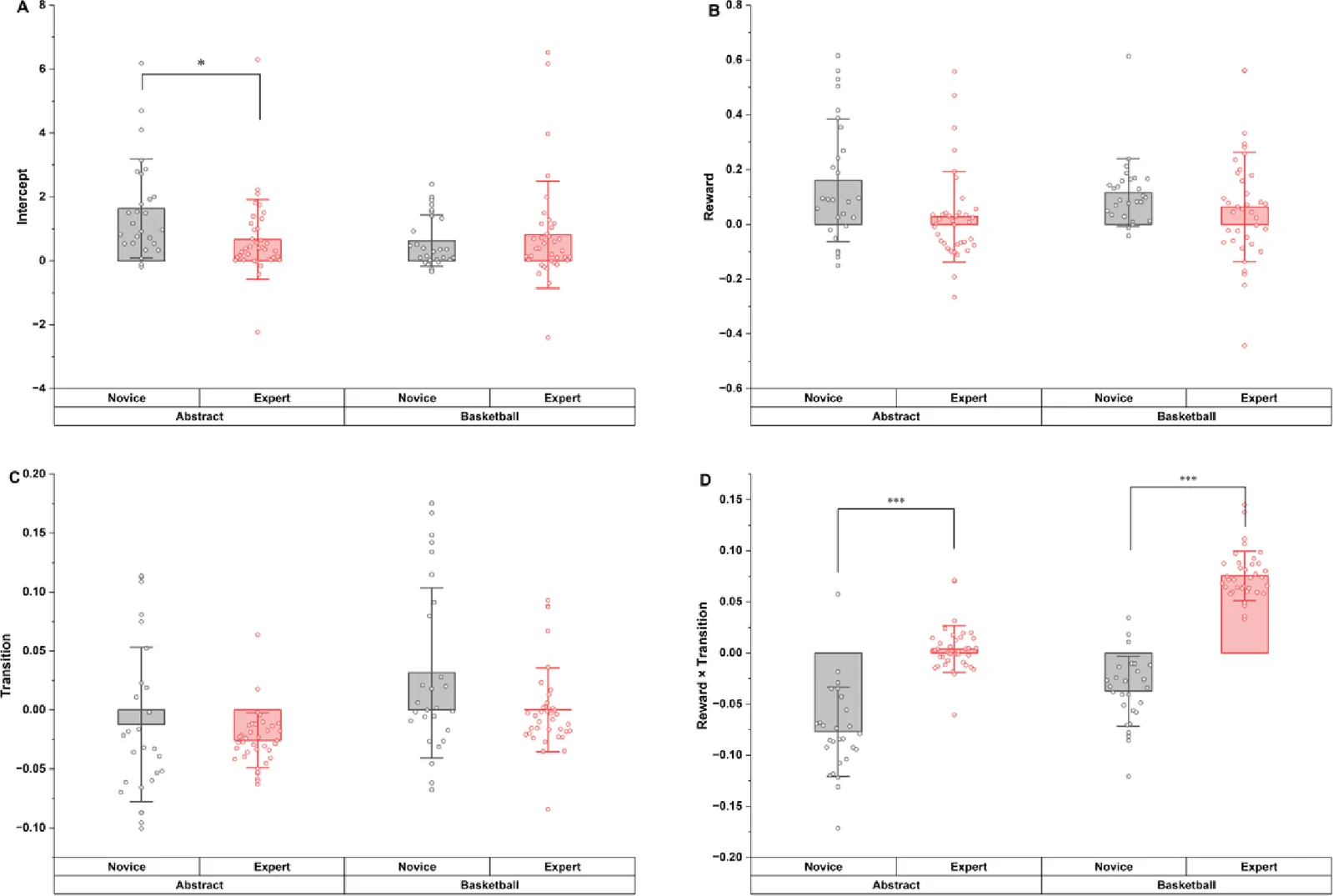
Coefficients of the logistic regression model. (**A**) The intercept coefficient of the participants in the abstract and basketball conditions. (**B**) The reward coefficient of the participants in the abstract and basketball conditions. (**C**) The transition coefficient of the participants in the abstract and basketball conditions. (**D**) The reward × transition coefficient of the participants in the abstract and basketball conditions. Each point represents the mean coefficient value for each participant. Error bars represent 1 SD.

The two-factor repeated measures ANOVA was conducted on the coefficients of the one-trial-back logistic regression model. For the intercept coefficient, the results revealed no significant main effect of Group, *F* (1, 62) = 1.87, *p* = 0.176, η^2^ = 0.020. A significant main effect of Condition was found, *F* (1, 62) = 4.69, *p* = 0.034, η^2^ = 0.024, the intercept was significantly higher in the abstract condition (M = 1.08, SD = 1.45) compared to the basketball tactical condition (M = 0.74, SD = 1.37). The interaction between Condition and Group was significant, *F* (1, 62) = 8.44, *p* = 0.005, η^2^ = 0.042. To further explore this interaction, simple effects analyses were conducted. In the abstract condition, novices (M = 1.64, SD = 1.54) showed significantly higher intercept values than basketball players (M = 0.67, SD = 1.25), *F* (1, 62) = 7.71, *p* = 0.014, η^2^ = 0.111. However, in the basketball tactical condition, no significant group difference was found, *F* (1, 62) = 0.28, *p* = 0.599, η^2^ = 0.004 (see Fig. 2a).

For the reward coefficient, the results revealed a significant main effect of Group, *F* (1, 62) = 6.43, *p* = 0.014, η^2^ = 0.061; novices (M = 0.14, SD = 0.18) showed higher reward sensitivity than basketball players (M = 0.05, SD = 0.18). No significant main effect of Condition was found, *F* (1, 62) = 0.02, *p* = 0.881, η^2^ < 0.001. The interaction between Condition and Group was not significant, *F* (1, 62) = 2.02, *p* = 0.160, η^2^ = 0.012 (see Fig. 2b).

For the transition coefficient, the results revealed a significant main effect of Group, *F* (1, 62) = 5.60, *p* = 0.021, η^2^ = 0.048; novices (M = 0.01, SD = 0.07) showed higher transition parameter than basketball players (M = − 0.01, SD = 0.03). A significant main effect of Condition was also found, *F* (1, 62) = 16.90, *p* < 0.001, η^2^ = 0.108; the transition parameter was higher in the basketball tactical condition (M = 0.01, SD = 0.06) compared to the abstract condition (M = − 0.02, SD = 0.05). The interaction between Condition and Group was not significant, *F* (1, 62) = 1.14, *p* = 0.290, η^2^ = 0.008 (see Fig. 2c).

For the reward × transition interaction coefficient, the results revealed a significant main effect of Group, *F* (1, 62) = 285.99, *p* < 0.001, η^2^ = 0.711; novices (M = − 0.06, SD = 0.04) exhibited lower reward × transition interaction values than basketball players (M = 0.04, SD = 0.04). A significant main effect of Condition was also observed, *F* (1, 62) = 107.65, *p* < 0.001, η^2^ = 0.448; the parameter was significantly higher in the basketball tactical condition (M = 0.03, SD = 0.06) compared to the abstract condition (M = − 0.03, SD = 0.05). The interaction between Condition and Group was also significant, *F* (1, 62) = 8.89, *p* = 0.004, η^2^ = 0.063. The simple effects analyses were conducted. In the abstract condition, novices (M = − 0.08, SD = 0.04) showed significantly lower reward × transition values than basketball players (M = 0.00, SD = 0.02), *F* (1, 62) = 92.70, *p* < 0.001, η^2^ = 0.599. Similarly, in the basketball tactical condition, novices (M = − 0.04, SD = 0.03) exhibited significantly lower values than basketball players (M = 0.07, SD = 0.02), *F* (1, 62) = 241.00, *p* < 0.001, η^2^ = 0.796 (see Fig. 2d).

### Reaction times

**Fig. 3:**
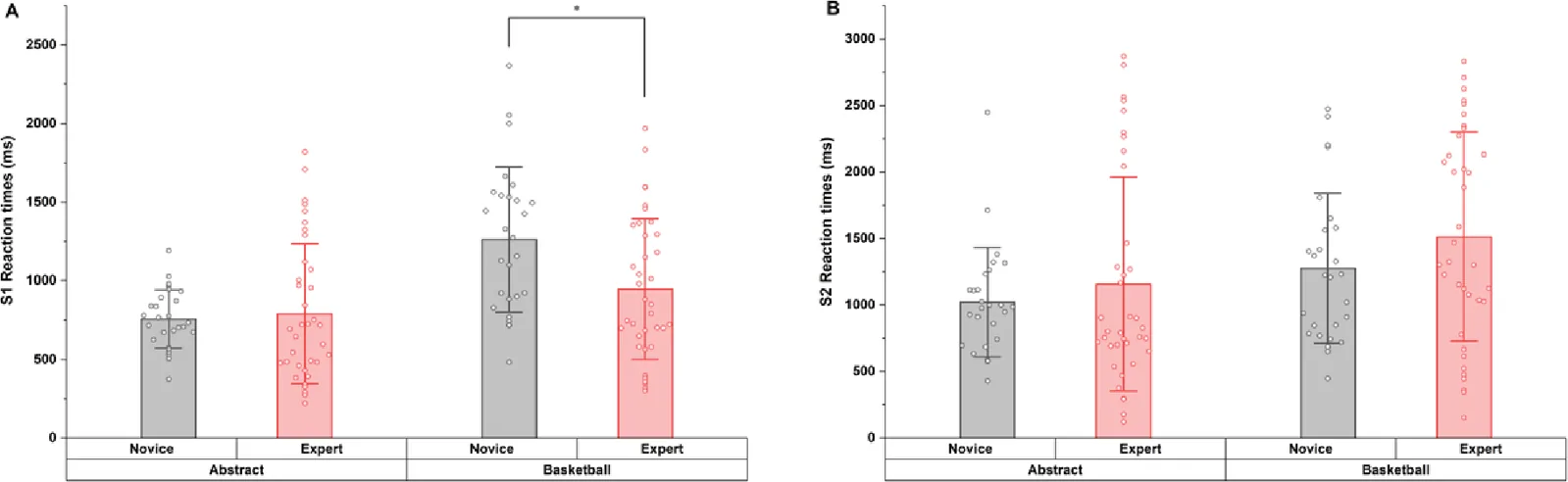
Results of the reaction times. (**A**) reaction times of the participants in S1. (**B**) reaction times of the participants in S2. Each point represents the mean reaction time for each participant. Error bars represent 1 SD. S1, stage one (i.e., the first stage); S2, stage two (i.e., the second stage).

The two-factor repeated measures ANOVA was conducted on the S1 and S2 reaction times. For the S1 reaction times, the results revealed no significant main effect of Group, *F* (1, 62) = 2.28, *p* = 0.136, η^2^ = 0.029. A significant main effect of Condition was found, *F* (1, 62) = 54.20, *p* < 0.001, η^2^ = 0.142; participants responded more slowly in the basketball tactical condition (M = 1080.89, SD = 476.28) compared to the abstract condition (M = 776.67, SD = 356.90). The interaction between Condition and Group was also significant, *F* (1, 62) = 15.06, *p* < 0.001, η^2^ = 0.044. The results of simple effects analyses revealed that: in the basketball tactical condition, novices (M = 1263.24, SD = 462.25) exhibited significantly longer reaction times than basketball players (M = 947.81, SD = 446.68), *F* (1, 62) = 7.56, *p* = 0.016, η^2^ = 0.109. However, in the abstract condition, the group difference was not significant, *F* (1, 62) = 0.14, *p* = 0.709, η^2^ = 0.002 (see Fig. 3a).

For the S2 reaction times, the results revealed no significant main effect of Group, *F* (1, 62) = 1.43, *p* = 0.236, η^2^ = 0.018. A significant main effect of Condition was found, *F* (1, 62) = 16.28, *p* < 0.001, η^2^ = 0.047; participants responded more slowly in the basketball tactical condition (M = 1412.64, SD = 706.21) compared to the abstract condition (M = 1101.11, SD = 666.21). The interaction between Condition and Group was not significant, *F* (1, 62) = 0.44, *p* = 0.508, η^2^ = 0.001 (see Fig. 3b).

### Subjective evaluations

The two-factor repeated measures ANOVA was conducted on the subjective evaluations. For the understanding, the results revealed a significant main effect of Group, *F* (1, 62) = 6.58, *p* = 0.013, η^2^ = 0.061; basketball players (M = 2.18, SD = 0.80) reported greater understanding than novices (M = 1.67, SD = 1.24). No significant main effect of Condition was found, *F* (1, 62) = 0.40, *p* = 0.528, η^2^ = 0.003. The interaction between Condition and Group was significant, *F* (1, 62) = 4.70, *p* = 0.034, η^2^ = 0.029.

**Fig. 4:**
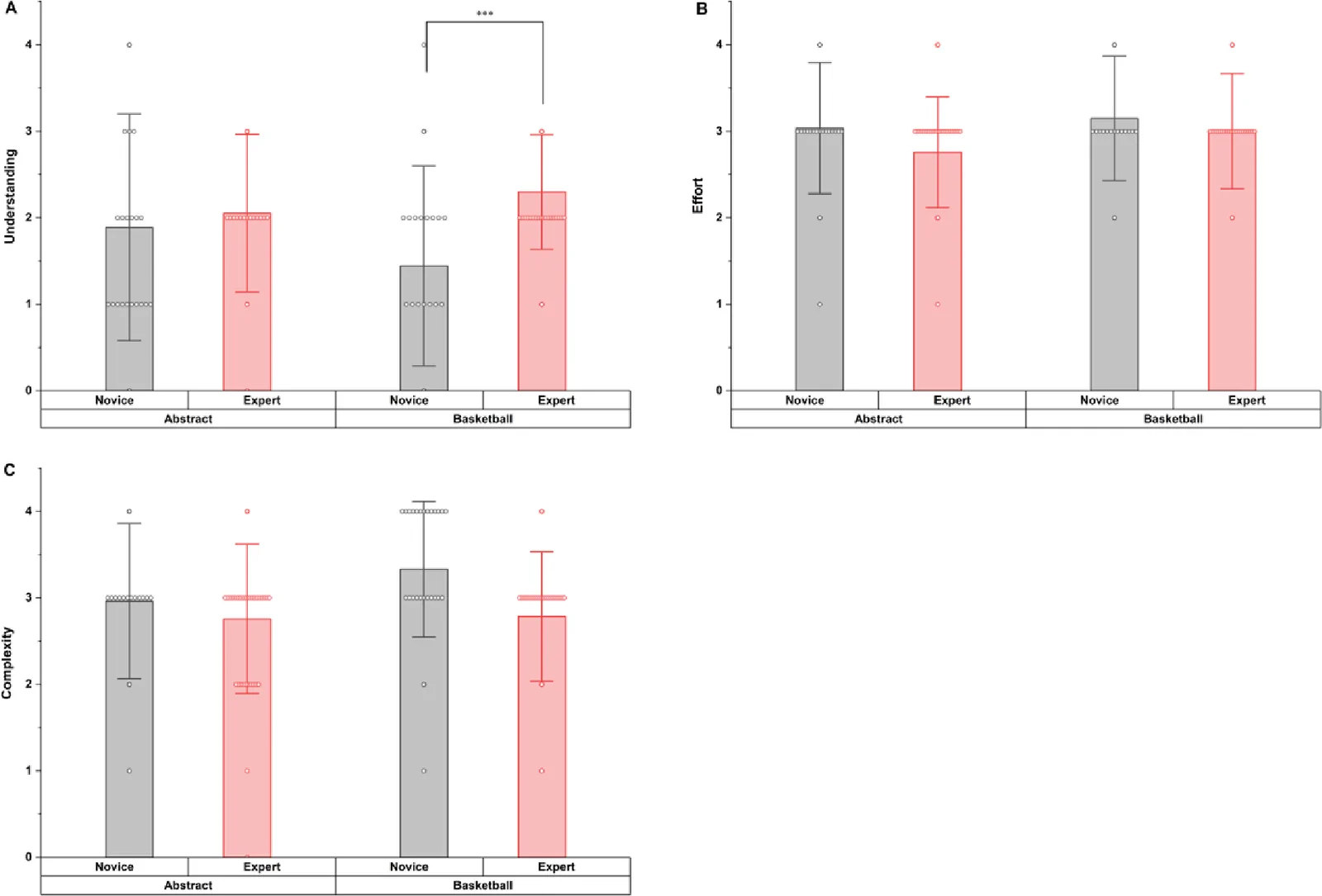
Results of the subjective evaluations. (**A**) The understanding of the participants in the abstract and basketball conditions. (**B**) The effort of the participants in the abstract and basketball conditions. (**C**) The complexity of the participants in the abstract and basketball conditions. Each point represents the subjective evaluation score for each participant. Error bars represent 1 SD.

The results of simple effects analyses revealed that, in the basketball tactical condition, basketball players (M = 2.30, SD = 0.66) reported significantly higher understanding than novices (M = 1.44, SD = 1.16), *F* (1, 62) = 14.00, *p* < 0.001, η^2^ = 0.184. However, in the abstract condition, the group difference was not significant, *F* (1, 62) = 0.35, *p* = 0.554, η^2^ = 0.006 (see Fig. 4a).

For the effort, the results revealed no significant main effect of Group, *F* (1, 62) = 2.00, *p* = 0.162, η^2^ = 0.024. A significant main effect of Condition was found, *F* (1, 62) = 4.12, *p* = 0.047, η^2^ = 0.016; participants reported greater effort in the basketball tactical condition (M = 3.06, SD = 0.69) compared to the abstract condition (M = 2.88, SD = 0.70). The interaction between Condition and Group was not significant, *F* (1, 62) = 0.57, *p* = 0.452, η^2^ = 0.002 (see Fig. 4b).

For the complexity, the results revealed a significant main effect of Group, *F* (1, 62) = 4.90, *p* = 0.031, η^2^ = 0.050; novices (M = 3.15, SD = 0.86) reported higher perceived complexity than basketball players (M = 2.77, SD = 0.80). No significant main effect of Condition was found, *F* (1, 62) = 2.77, *p* = 0.101, η^2^ = 0.014. The interaction between Condition and Group was not significant, *F* (1, 62) = 2.06, *p* = 0.156, η^2^ = 0.011 (see Fig. 4c).

## Discussion

The present study integrated the RL framework with the two-stage task to examine whether domain-specific contexts enhance MB decision-making among basketball players. By replacing the original abstract symbols with basketball tactical diagrams, we found that: (1) basketball players exhibited a larger reward × transition interaction and reported higher task understanding in the basketball condition than in the abstract condition, supporting Hypotheses 1 and 3a; (2) participants demonstrated significantly longer reaction times in both S1 and S2, supporting Hypothesis 2; (3) participants reported greater effort in the basketball condition, providing no support for Hypotheses 3b; and (4) basketball players perceived the task as equal complex cross basketball-specific and abstract contexts, supporting Hypothesis 3c. By bridging the gap between basketball games and the two-stage task, the basketball tactical diagrams we designed not only mirrored real-competition scenarios, but also adhered to naturalistic RL^30^.

The nature of the task plays a pivotal role in determining the relative contributions of MF and MB systems, highlighting the complex interplay among expertise, context, and problem specificity^2^. Transforming abstract symbols into basketball tactical diagrams encouraged basketball players to use a more MB decision-making strategy^19^. This underscores the principle that the strategies behind expert decisions are contingent upon the immediate characteristics of the environment and the specific stimuli presented^31^. In the sports domain, elite referees demonstrated superior decision-making performance when assessed using video clips that closely aligned with the refereeing^6^. In contrast, in a task unfamiliar to both athletes and non-athletes, the expertise advantage disappears^25^. These patterns illustrate that expertise benefits emerge primarily in domain-relevant contexts, consistent with our hypothesis that basketball players would show a stronger MB tendency in the basketball-specific condition. Decision-making processes in humans exhibit dynamic progression. Initially, individuals may depend on trial-and-error tactics; with increasing expertise, they gradually construct a conceptual model of the task’s structure and dynamics, enabling a shift toward MB decision-making^32^. Furthermore, providing explicit information about task structure strongly boosted the influence of MB RL, this effect aligns with meta-cognitive cost-benefit decision-making processes^33^. An accurate understanding of the task structure enhances the precision of MB predictions and increases the expected benefits of MB strategies^5,17^. In our study, the instructions provided before each block were abstract and obscure, whereas the tactical diagrams implicitly evoked the participants’ own expertise related to basketball training and competitions. This expertise likely provided guidance for performing the two-stage task and facilitated MB strategies without the need for explicit structural instruction^34,35^.

Beyond the reward × transition interaction, the reward and transition coefficients further illuminate how expertise shapes decision-making strategies. Firstly, novices showed a higher reward coefficient than basketball players, indicating a stronger tendency to repeat actions following rewards, which is a hallmark of MF decision-making. It suggests that compared to basketball players, novices rely more on immediate reinforcement rather than integrating structural information into their choices. Previously research indicates that experts rely less on short-term outcome feedback and more on an internalized representation of environment to guide behavior^36,37^. Secondly, novices also exhibited a higher transition coefficient compared to basketball players. Although to some extent a higher transition coefficient generally indicates better utilization of the probabilistic transition structure of the task when making S1 choices, the reward × transition interaction is the direct indicator of the defining characteristic of model-based decision-making^5^. Extensive domain-specific training enables experts to develop stable internal models, allowing them to integrate reward and transition rather than reacting to single instances of feedback or transition^38^. Moreover, even in the absence of reward information, MB decision-making via state prediction error can still be present^39^. Taken together, the expertise advantage is characterized by the ability to integrate feedback with the structural features of the environment, and this ability generalizes beyond expertise-relevant condition^40^.

Although the basketball tactical and abstract conditions shared identical underlying task structures, the basketball tactical diagrams contained a greater amount of information. This increased information likely required participants to engage in deeper cognitive processing, resulting in longer reaction times and self-reported effort. For basketball players, this is consistent with the results of Shahar et al.^21^, which demonstrated a positive correlation between reaction times and parameters reflecting MB behavior, implying that MB strategy is a cognitive resource-intensive and time-consuming process. In contrast, novices also exhibited prolonged reaction times and greater effort in the basketball tactical condition, yet did not show a corresponding increase in the reward × transition interaction. This may indicate that greater engagement alone is insufficient to elicit MB strategy in the absence of relevant domain knowledge and experience. Therefore, we are able to speculate that the MB strategy is the reason for the longer reaction times, but longer reaction times do not necessarily lead to the emergence of MB strategy.

Given that the basketball and abstract conditions shared identical task structures, the basketball and abstract conditions shared identical task structures, the experts did not perceive the basketball-specific context as less complex than the abstract. Novices reported higher overall perceived complexity than basketball players, suggesting that expertise may help attenuate cognitive demands across contexts. This aligns with the findings of Feher da Silva et al.^17^, who demonstrated that story instructions fostered MB behavior and improved understanding, but did not reduce perceived complexity. In the present study, the absence of explicit instructions, combined with novices’ lack of basketball-related expertise to guide decision-making, may explain why they did not exhibit the same MB tendency as basketball players^41^. This pattern further supports the notion that the decision-making advantages of experts can generalize across different task contexts.

The present study implements a basketball-specific adaptation of the two-stage task and directly compares it with the original abstract condition to evaluate the impact of domain-specific context on decision-making strategies. In doing so, it extends the application of RL from general psychological research to sport psychology. By incorporating basketball tactical diagrams, this study improves ecological validity and better reflects decision-making demands in real-world sport contexts.

However, several limitations of this study are as follows. First, the study was confined to basketball, necessitating further exploration in other sports and fields to validate the applicability of our findings. Second, while the two-step task offers a controlled framework to investigate decision strategies, it may not fully capture the dynamic nature of real sports scenario. Future studies should consider more ecologically valid task designs. Finally, our methodological approach is primarily behavioral. Future studies could benefit from integrating neuroscientific techniques such as electroencephalography (EEG) and functional magnetic resonance imaging (fMRI), to delve into the neural mechanisms of decision-making in sports contexts.

## Conclusion

In conclusion, this study suggests that basketball players rely more on MB decision strategies across basketball and abstract contexts, underscoring the importance of expertise in shaping decision making. MB strategies tend to produce longer reaction times, yet long reaction times do not necessarily indicate the use of MB strategies. Based on our findings, coaches and trainers should be encouraged to incorporate domain-specific contexts into their training plans to improve athletes’ decision-making skills.

## Data Availability

The data supporting the findings of this study are available upon request. Please contact the corresponding author for data access inquiries.
